# Oxygen limitation modulates pH regulation of catabolism and hydrogenases, multidrug transporters, and envelope composition in *Escherichia coli *K-12

**DOI:** 10.1186/1471-2180-6-89

**Published:** 2006-10-06

**Authors:** Everett T Hayes, Jessica C Wilks, Piero Sanfilippo, Elizabeth Yohannes, Daniel P Tate, Brian D Jones, Michael D Radmacher, Sandra S BonDurant, Joan L Slonczewski

**Affiliations:** 1 Department of Biology, Kenyon College, Gambier, OH 43022, USA; 2 Department of Mathematics, Kenyon College, Gambier, OH 43022, USA; 3 Gene Expression Center, University of Wisconsin, Madison, WI 53706, USA

## Abstract

**Background:**

In *Escherichia coli*, pH regulates genes for amino-acid and sugar catabolism, electron transport, oxidative stress, periplasmic and envelope proteins. Many pH-dependent genes are co-regulated by anaerobiosis, but the overall intersection of pH stress and oxygen limitation has not been investigated.

**Results:**

The pH dependence of gene expression was analyzed in oxygen-limited cultures of *E. coli *K-12 strain W3110. *E. coli *K-12 strain W3110 was cultured in closed tubes containing LBK broth buffered at pH 5.7, pH 7.0, and pH 8.5. Affymetrix array hybridization revealed pH-dependent expression of 1,384 genes and 610 intergenic regions. A core group of 251 genes showed pH responses similar to those in a previous study of cultures grown with aeration. The highly acid-induced gene *yagU *was shown to be required for extreme-acid resistance (survival at pH 2). Acid also up-regulated fimbriae (*fimAC*), periplasmic chaperones (*hdeAB*), cyclopropane fatty acid synthase (*cfa*), and the "constitutive" Na+/H+ antiporter (*nhaB*). Base up-regulated core genes for maltodextrin transport (*lamB*, *mal*), ATP synthase (*atp*), and DNA repair (*recA*, *mutL*). Other genes showed opposite pH responses with or without aeration, for example ETS components (*cyo*,*nuo*, *sdh*) and hydrogenases (*hya, hyb, hyc, hyf, hyp*). A *hypF *strain lacking all hydrogenase activity showed loss of extreme-acid resistance. Under oxygen limitation only, acid down-regulated ribosome synthesis (*rpl*,*rpm*, *rps*). Acid up-regulated the catabolism of sugar derivatives whose fermentation minimized acid production (*gnd*, *gnt*, *srl*), and also a cluster of 13 genes in the *gadA *region. Acid up-regulated drug transporters (*mdtEF*, *mdtL*), but down-regulated penicillin-binding proteins (*dacACD*, *mreBC*). Intergenic regions containing regulatory sRNAs were up-regulated by acid (*ryeA*, *csrB*, *gadY*, *rybC*).

**Conclusion:**

pH regulates a core set of genes independently of oxygen, including *yagU*, fimbriae, periplasmic chaperones, and *nhaB*. Under oxygen limitation, however, pH regulation is reversed for genes encoding electron transport components and hydrogenases. Extreme-acid resistance requires *yagU *and hydrogenase production. Ribosome synthesis is down-regulated at low pH under oxygen limitation, possibly due to the restricted energy yield of catabolism. Under oxygen limitation, pH regulates metabolism and transport so as to maximize alternative catabolic options while minimizing acidification or alkalinization of the cytoplasm.

## Background

Both pH and oxygen are important factors governing bacterial growth. Acid and base regulate many genes and proteins in *Escherichia coli *and related enteric bacteria [1-5]. Oxygen limitation regulates numerous genes such as those of the FNR and ArcA regulons [6,7]. Some genes are known to be coinduced by acid and low oxygen, such as the amino-acid decarboxylases [8-10], whereas others are coinduced by base and low oxygen [5,11]. For many genes, however, regulation has been characterized only with respect to pH or to oxygen, not for both factors. Transcriptomic studies of pH stress have focused mainly on aerated cultures [2,12].

The intersection of two stress factors is rarely addressed in global responses studies. An exceptional example is Kustu's study of nitrogen and sulfur starvation in *E. coli *[13,14], which reveal unexpected intersections of response; for example, while the RpoS regulon is induced for both nitrogen and sulfur starvation, certain elements of the regulon are induced under sulfur starvation but repressed under nitrogen starvation. The intersection of stress is important because natural environments show complex interaction of stress conditions. For example, *Salmonella typhimurium *grown intracellularly within macrophages show a protein profile very different from the protein profiles for isolated stresses such as acid stress and oxidative stress [15]. The intersection of stress responses is highly relevant to bacterial growth under natural and medically relevant conditions.

Acid and base stress are key factors of the enteric environment. Bacteria grow and persist in the intestine within a moderate range of external pH 5–8 [16], but colonization requires transient survival through the stomach at pH 1–4 [17] and subsequent exposure to pancreatic secretions at pH 10 [18]. Growth of *E. coli *at moderately low or high pH levels (pH 5 to 6 or pH 8 to 9, respectively) induces protective responses that maintain internal pH homeostasis near pH 7.6 [19], and prepare the cell to survive future exposure to more extreme pH conditions that no longer permit growth [20,21]. For example, growth in acid down-regulates the transport and catabolism of carbon sources whose breakdown generates excess acids [22]. Growth at high pH increases proton uptake and minimizes proton export [2], Survival in extreme acid, either constitutive or up-regulated by moderate acid, is a key trait of gastrointestinal pathogens [23]. Specific virulence factors, such as ToxR-ToxT in *Vibrio cholerae *[24] and the pH 6 antigen of *Yersinia pestis *[25], are up-regulated by acid. Acid stress also has important protective applications, for example contributing to food preservation by amplifying uptake of organic acids [26,27].

Processes leading to either acidification or alkalinization commonly coincide with low oxygen. Acid and anaerobiosis co-induce the catabolic decarboxylases for lysine and arginine [8-10]; the hydrogenases Hyd-1 [28], Hyd-4 [29,30], and formate-lyase complex FHL [31]; catabolic enzymes such as ManX and GapA [5,11]; transporters such as the nickel transporter NikA [5]; and periplasmic proteins TolC and HdeA [5]. Base and anaerobiosis up-regulate glutamate dehydrogenase [11]; the deaminases for tryptophan and serine [5,11]; and periplasmic proteins such as ProX, OppA, and DegQ [5]. Furthermore, responses commonly associated with "stationary phase," such as RpoS induction, often involve low-oxygen conditions combined with pH increase [32] or accumulation of fermentation acids such as acetate [33-36].

We used microarrays to survey pH-dependent gene expression in *E. coli *cultured under oxygen-limited conditions. Our experimental design enabled comparison with our previous study of pH-dependent genes in well aerated cultures. Our new study reveals patterns of pH response that require oxygen limitation, as well as pH responses that are independent of oxygen.

## Results

### Oxygen-limited growth as a function of pH

*E. coli *W3110 was cultured in LBK buffered with a dibasic acid, HOMOPIPES, which provides good buffering capacity at both low and high pH [2]. Oxygen limitation was achieved using growth in closed tubes, as conducted previously [5,11,37], under conditions that avoid the CO_2 _depletion that occurs during flushing with inert gases [37]. Bacteria were cultured in medium buffered at pH 5.7, pH 7.0, and pH 8.5 respectively. The high and low pH values were chosen so as to achieve reproducible and comparable rates of growth. Both pH values are closer to neutrality than those used for aerated cultures [2], because the pH range for growth is narrower under oxygen limitation than it is for aerated cultures [11]. A stationary-phase culture from unbuffered LBK medium was diluted into each of the three buffered media, and incubated until OD_600_= 0.2. Growth was logarithmic throughout this period, through approximately four to five doublings. The growth rates observed were 1.8 generations per hour (pH 7.0), 1.3 gen/h (pH 5.7), and 1.1 gen/h (pH 8.5), with uncertainty estimated at ± 0.2 gen/h. For array hybridization, our experimental design and analysis were consistent with the "consensus" recommendations of Allison [38] in that we included an ample number of biological replicates (five independent cultures for each growth condition), assuring high power of detection as well as a low false-positive rate.

### Analysis of expression ratios

The cDNA from five independent cultures of each pH condition were hybridized to Affymetrix antisense *E. coli *arrays. Array data have been deposited at the NCBI Gene Expression Omnibus (accession GSE4556). For comparison, the array data from aerated cultures [2] are available (GSE4511).

**Figure 1:**
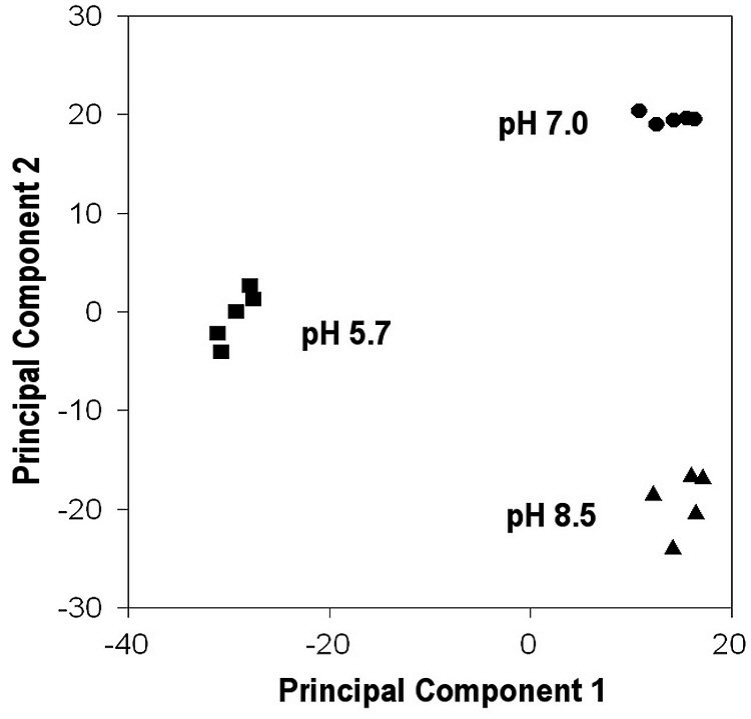
**Principal components analysis**. The gene expression profiles of the arrays were visualized in two-dimensional Euclidian space, by using BRB ArrayTools software. The horizontal axis represents the first principal component of variation, that is, a projection of the array data representing the maximal variance among them. The vertical axis represents the second principal component of variation. The pH 5.7, squares; pH 7.0, circles; pH 8.5, triangles.

The basis of variation in expression among the fifteen anaerobic cultures was tested globally by a principal components analysis of the expression indices determined by Affymetrix chip hybridization (Fig. 1). The principal components analysis transforms the data to a coordinate system in which the major part of the variation in the dataset lies along one axis, the first principal component; then the next greatest part of variation lies along the second axis, the second principal component of variation. Plotting the array data on these principal component axes of maximal variation allows the maximal separation of the data in two-dimensional space, and therefore aids in differentiating among the experimental trials.

Over the first two components of variation, the array hybridization signals (converted to expression indices) showed three well-separated groups, each identified with one of the pH conditions. Thus, the majority of variation among the arrays was clearly associated with the pH of the growth medium. The only other difference among these three cultures was the range of potassium concentration, which varied slightly with pH adjustment (approximately 150 mM–250 mM K^+ ^in buffered LBK). As in our previous microarray study using the same culture medium [2], we saw no evidence of K^+^-dependent gene expression.

The principal components analysis indicates that our experiment successfully distinguished growth on the basis of pH. Furthermore, in our gene-by-gene comparison, many members of a given operon showed parallel profiles of expression among the three pH conditions, an indication that our observed expression ratios are consistent and biologically relevant.

To assess statistical significance of bacterial expression ratios, several different statistical methods are now used, such as a fold-change significance threshold [39], ranked t-tests [40], and Bayesian statistics [7,41]. We chose the gene-by-gene ANOVA with Tukey's correction as a conservative and appropriate choice for comparing three experimental groups [42].

The expression indices for each gene and intergenic region among the three pH groups were compared using one-way ANOVA at a significance level of 0.001 [2,42]. This means that approximately one false positive would be expected per thousand genes tested, or approximately seven false positives out of the Affymetrix gene set. A total of 1,384 genes and 610 intergenic regions (IG) showed at least one significant acid/base expression ratio (pH 5.7/pH 7.0; pH 7.0/pH 8.5; and/or pH 5.7/pH 8.5). The full list of expression indices and pH-dependent expression ratios are compiled in Additional file 1 (genes) and Additional file 2 (intergenic regions). 182 genes showed pH-dependent expression ratios of 4-fold or greater (log_2 _ratios ≥ 2); these are presented in Table 1.

Note that throughout our report (Tables 1, 3, 4, and Additional file 3), the three classes of expression ratios are presented as the quotient "acid/base" so that the log_2 _value of the ratio is positive for expression increased in acid (or decreased in base), and negative for expression increased in base (or decreased in acid). The ratio pH 5.7/pH 8.5 was used to designate genes as "acid up-regulated" or "base up-regulated." The terms "up-regulated" and "down-regulated" refer only to ratios of RNA abundance, without implying a regulatory mechanism [39,40]. Values presented in bold font indicate significance based on Tukey's test, p ≤ 0.001.

Of the most highly acid-up-regulated genes under oxygen limitation (Table 1), only 20% appear previously as acid-up-regulated under aeration [2]. Of the genes up-regulated by base under oxygen limitation, 20% are up-regulated in base under aeration, but also 10% are upregulated in acid under aeration [2]. Overall, oxygen limitation had a substantial impact on the profile of pH-regulated genes.

The exposure of a given protein to external pH depends on cell location, in that the cytoplasmic pH is maintained near pH 7.6 in growing cells, whereas the outer membrane, periplasm, and periplasmic face of the inner membrane are exposed to external pH. The numbers of pH-dependent genes in these subcellular locations were compared. (Both aerobic [2] and oxygen-limited conditions were included in the totals compared.) pH-dependent expression was observed for 50% of genes encoding known periplasmic proteins, as compared to 47% of inner membrane proteins, 42% of outer membrane proteins, and 39% of non-ribosomal cytoplasmic proteins. Thus the protein composition of the periplasm appeared to be the most sensitive to external pH, wherease the cytoplasmic protein composition was the least pH-sensitive.

### Core pH stress genes

A set of genes were identified that showed pH-dependent expression under oxygen limitation (Table 2) as well as reported previously under aeration [2]. These were designated "core pH stress genes." A quarter of these core pH stress genes as yet have no known function, such as the highly acid-inducible membrane protein *yagU*, which showed the sixth-highest acid/base expression ratio (pH 5.7/pH 8.5) in Table 1.

The *yagU *gene encodes an uncharacterized protein putatively assigned to the inner membrane [43,44]. We transduced a *yagU*::*kan*^R ^allele from the Blattner collection [45] into our acid-resistant strain W3110. The *yagU *construct showed 3-fold lower acid resistance than the parent strain (19 ± 2% survival at pH 2, compared to 70 ± 6% for W3110).

Catabolic operons for sugar alcohols galactitol (*gat*) and sorbitol (*srl*) were both up-regulated in acid. Other genes of known function showing a high acid/base expression ratio included fimbriae (*fimAC*), periplasmic chaperones (*hdeAB*), cyclopropane fatty acid synthase (*cfa*), the "constitutive" Na+/H+ antiporter (*nhaB*), and about thirty unidentified proteins. Core pH genes up-regulated at high pH included maltodextrin transport (*lamB*, *mal*), ATP synthase (*atp*), envelope stress (*cpxPR*), and DNA repair (*recA *and *mutL*). These are consistent with previous reports of regulation (in aerated cultures) of *mal *[22], ATP synthase [2], *cpxR *[46], and SOS DNA repair [47].

Other genes systems showed oppositely directed acid/base responses in anaerobic versus aerobic conditions. In particular, several components of electron transport and the TCA cycle were acid-repressed under oxygen limitation, though up-regulated in acid with aeration [2]. These include the *ace*, *cyo*, *nuo*, *sdh*, and *suc *operons. On the other hand, the hydrogenase gene *hybA *showed the opposite pattern (up in acid, anaerobically; in base, aerobically).

### Acid/base expression ratios confirmed by real-time PCR

**Figure 2:**
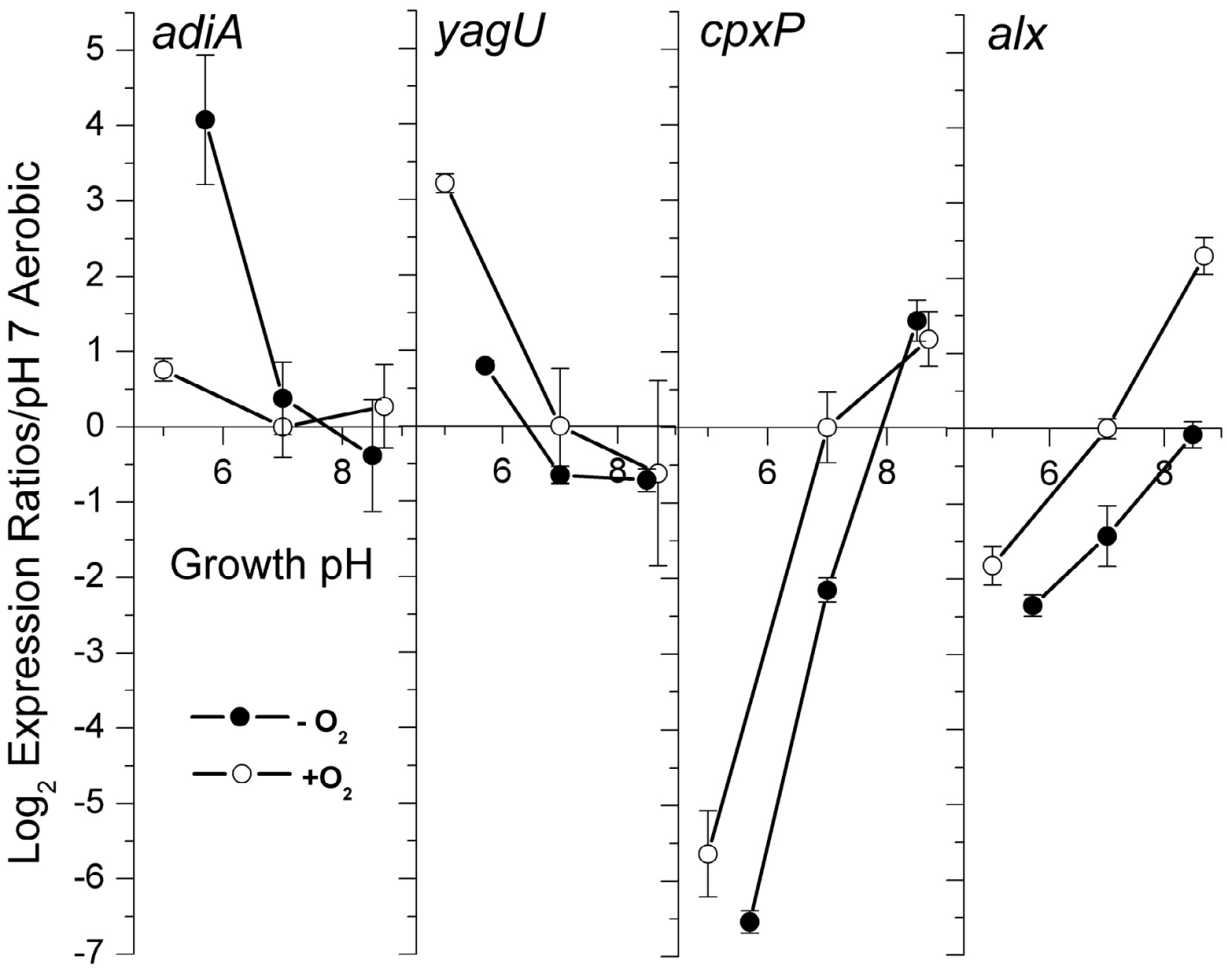
**Real-time PCR expression ratios of selected genes**. Bacteria were cultured in acid or base, or at pH 7.0, with aeration (○) or under oxygen limitation (●) (see Methods). For each gene, expression levels are shown for amplified message in acid or base, relative to the level at pH 7 with aeration.

The pH dependence of representative genes was confirmed by real-time PCR of cultures grown under anaerobic conditions, as well as aerobic conditions based on Ref. [2] (Fig. 2). The real-time PCR expression ratios are shown for up-regulation in acid or in base, relative to expression at pH 7. For example, the gene encoding arginine decarboxylase, *adiA*, showed strong induction in acid under anaerobic conditions but showed no significant expression aerobically. These real-time PCR data are consistent with our array data, and with the known coinduction of *adiA *by acid and anaerobiosis [8]. The *yagU *gene showed strong increase by acid both aerobically and anaerobically; this result was also consistent with the arrays.

The envelope stress protein *cpxP *[46] and the base-inducible tellurium resistance homolog *alx *[5,48] showed mRNA levels increased at high pH. High-pH up-regulation was seen both aerobically and anaerobically. For *alx*, repression by acid below pH 7 was seen only under aerobic, not anaerobic conditions; this pattern was seen in the array data (Table 1, and Ref. [2]) as well as in the real-time PCR (Fig. 2).

### Hydrogenases regulated by pH

*E. coli *fermentation generates substrates for hydrogenase enzymes, which interconvert hydrogen ions with hydrogen gas [49-51] and generate H_2 _from formate through association with formate dehydrogenase [31,52,53]. Several hydrogenases of *E. coli *respond to pH and oxygen level [31,54], although the overall pattern remains unclear, especially at high pH.

**Figure 3:**
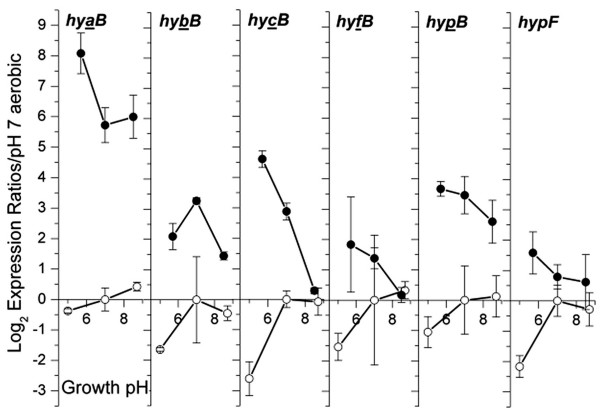
**Hydrogenase genes: Real-time PCR expression ratios (aerobic and oxygen-limited)**. Expression levels were determined as for Fig. 2.

We sought to clarify the pattern of expression of all the hydrogenases (*hya, hyb, hyc, hyf*) as well as hydrogenase assembly (*hyp*) as a function of pH. Real-time PCR was used to measure expression of one gene from each of the hydrogenase operons (Fig. 3). The log expression of each gene is normalized to its expression level at pH 7 with aeration. Under oxygen limitation, each hydrogenase operon showed higher expression in acid than in base. During growth with aeration, however, the acid/base effect was reversed: Each gene showed higher expression in base than in acid. The up-regulation at high pH was particularly strong for *hycB *(6-fold greater at pH 8.7 than pH 5.0) and *hypF *(4-fold).

Hydrogenase activity may be important at low pH for its contribution to expulsion of excess protons from the cytoplasm [49,55,56]. The importance of hydrogenase expression for pH stress was confirmed by the loss of acid resistance in a strain defective for *hyp*. We transduced a *hypF*::*kan *allele (strain provided by K. T. Shanmugam) into our W3110 strain. The *hypF *defect abolished all hydrogenase activity as tested by methylviologen assay. The effect of *hypF *on acid resistance was tested for cultures grown at pH 5 to stationary phase, an oxygen-limited condition in which acid resistance (survival at pH 2) is induced [21]. The *hypF *defect decreased stationary-phase acid resistance to less than 3%, about 20-fold lower than the parent strain. No effect on acid survival was seen, however, in a strain defective for a single hydrogenase operon (*hya*, *hyb*, or *hyc*). Thus, hydrogenase activity by one or more of the hydrogenase systems was necessary for stationary-phase acid resistance.

### Ribosome synthesis depressed by acid

Ribosome synthesis is found to be down-regulated under conditions in which energy yield is restricted, such as carbon starvation [39] or nitrogen and sulfur starvation [40]. We found extensive down-regulation of virtually all genes encoding ribosome subunits during growth in acid under oxygen limitation (*rpl*,*rpm*, *rps*) (see Additional file 1). None of these genes show a significant effect of pH under aeration [2].

### Catabolism regulated by pH

In aerated cultures, acid up-regulates genes for transport and catabolism of sugars and sugar derivatives outside the glucose pathways: ribose (*rbs*), galactitol (*gat*), sorbitol (*srl*, *gut*), and gluconate (*gnd*) [2]. Under oxygen limitation, acid up-regulated additional catabolic enzymes and transporters for arabinose (*ara*), fuculose (*fuc*), gluconate (*gnt*), mannitol (*mtl*), and melibiose (*mel*) (see Additional file 3). At high pH, however, there was strong up-regulation of fructose catabolism (*fruABKR*) and the maltose regulon (*mal*) which breaks down maldodextrins to glucose. The acid up-regulation of *gat *[11] and the high-pH up-regulation of *mal *[22] were reported previously; regulation of the other catabolic systems was new to this report.

**Figure 4:**
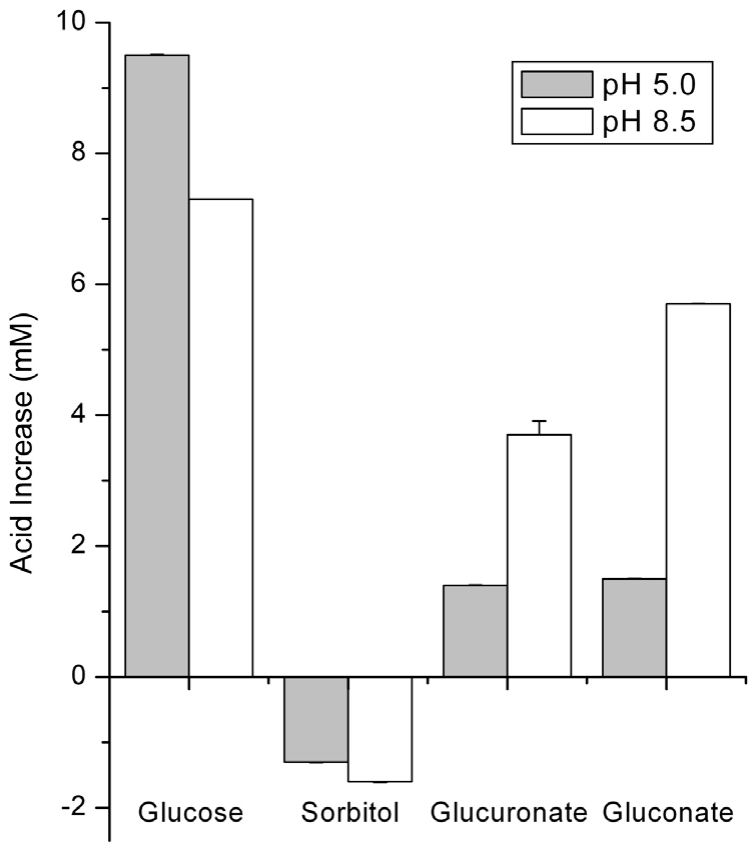
**Acid production during supplementation with different carbon sources**. The net acid produced or consumed by each culture was determined as described (see Methods).

Fermentation of sugar alcohols [57] and sugar acids [58] may cause less acidification than does glucose fermentation. It was proposed that the pH-dependent selection of sugar substrates may be correlated with their relative degree of acidification of the growth medium. The net acidification of media during catabolism of various substrates was tested (Fig. 4). *E. coli *strain W3110 was grown anaerobically to stationary phase on half-strength LBK medium supplemented with different carbon sources: glucose, sorbitol, gluconate, and glucuronate. Glucose fermentation was associated with the largest degree of acidification. By contrast, sorbitol-supplemented medium showed a net increase in pH, whereas the sugar-acids showed relatively small acidification. The difference between glucose and the other supplemental substrates was especially pronounced at the lower starting pH (pH 4.8) where acidification must be limited to allow growth.

Several amino acid decarboxylase operons that showed exceptionally high acid/base ratios (Table 1) are known to be up-regulated by acid with anaerobiosis: the degradative lysine decarboxylase, *cadBA *[8,10]; arginine decarboxylase, *adiAYC *[8,9,59]; and the glutamate decarboxylases, *gadAXW *and *gadBC *[20,60,61]. Under oxygen limitation, the *gad *genes also turned out to be among those most highly up-regulated at high pH (pH 8.5) compared to pH 7.0 (Table 1; Table 3). This is consistent with our previous reports that *gadA *and *gadBC *are up-regulated at high pH under anaerobiosis [5,11,62]. The entire series of genes in the vicinity of *gadAXW *(thirteen in all) showed the same pattern of induction at both low and high pH (Table 3), including the small RNA regulator *gadY *[63]. This "pH stress region" included genes encoding the *mdtEF *multidrug transporter [64,65] and the periplasmic acid-inducible chaperones *hdeAB *[66] as well as genes of unknown function, such as the OMP gene *sly*.

At high pH, deamination of amino acids is favored due to removal of ammmonium ion and production of fermentation acids [11]. Under oxygen limitation, at high pH, the amino acid deaminases and transporters were up-regulated (see Additional file 3): tryptophanase, *tnaAB*, and serine deaminase, *serABC *and transporters for other amine-rich molecules: arginine (*art*) and spermidine/putrescine (*pot*). The *tna *and *ser *results are consistent with previous studies based on proteomics and *lac *fusions[5,11]. The transport and interconversion of polyamines associated with amino-acid catabolism (*spe*, *pot*) was up-regulated at high pH. This finding is consistent with the report that polyamine stress in bacteria is amplified at high pH [67].

### Multidrug resistance and ion transporters

**Figure 5:**
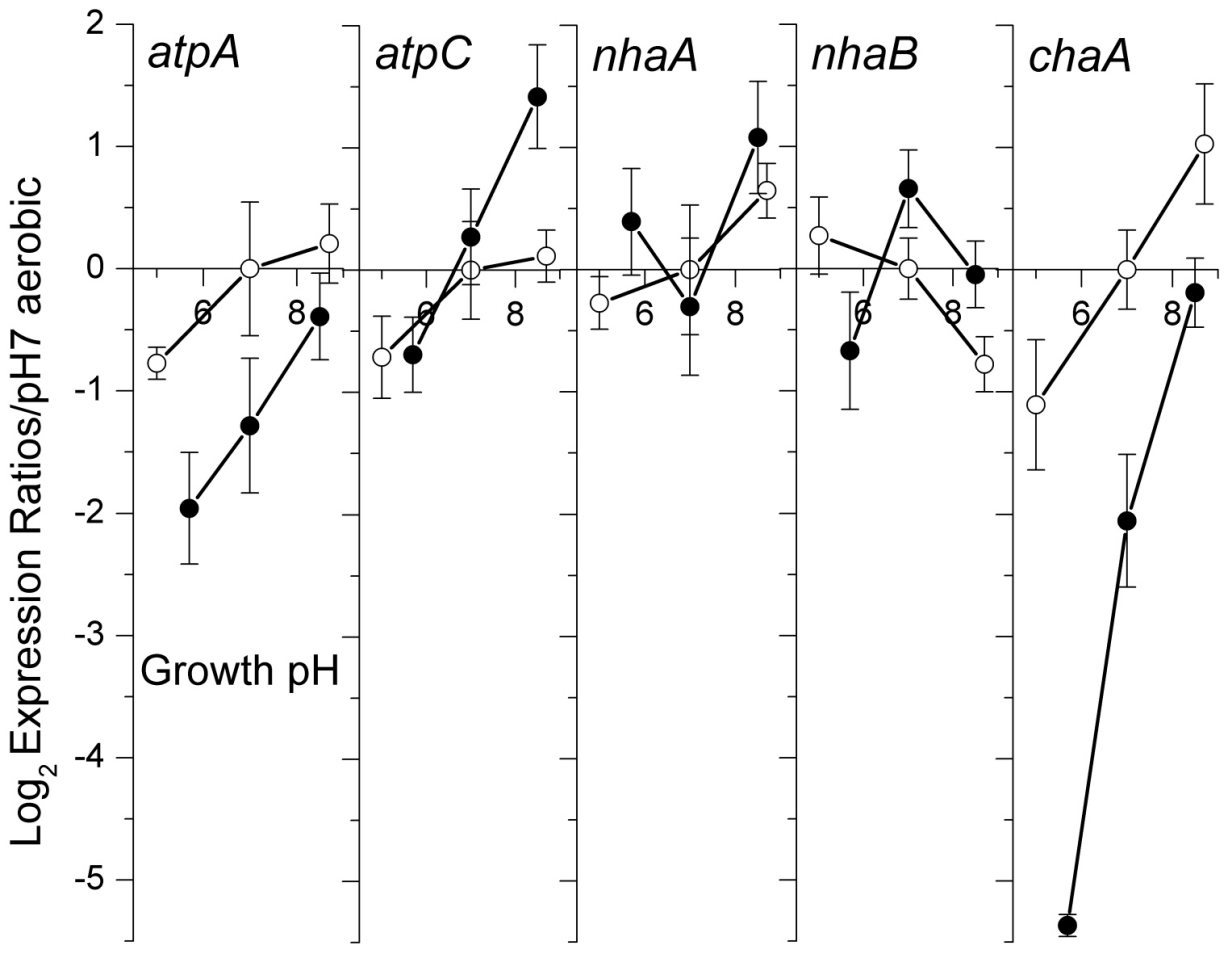
**Multiple-drug and ion transporters: Real-time PCR expression ratios (aerobic and oxygen-limited)**. Expression levels were determined as for Fig. 2.

Several ion transporters and multidrug resistance genes showed significant acid/base expression ratios under anaerobiosis (Table 4), some of which were confirmed by real-time PCR (Fig. 5). Nickel transport was increased by acid, consistent with our previous proteomic studies [5] and with the nickel requirement for hydrogenase activity up-regulated by acid under anaerobiosis [68]. Additionally, growth in acid enhanced the expression of transporters for copper, silver, iron, and magnesium (*copA*, *cusC*, *feoAB*,*mgtA*,).

High pH up-regulated several genes in the ATP synthase operon (*atp*) [69], as confirmed by real-time PCR (Table 4; Fig. 5). The increased production of ATP synthase compensates for decreased proton-motive force at high pH [2]. High pH also was associated with up-regulation of the Ca^2+^/H^+ ^antiporter *chaA *(Fig. 5), while its regulators *chaBC *were down-regulated. The *chaA *antiporter is known to be up-regulated at high pH where it extrudes sodium ion [70]. High pH also elevated expression of acridine efflux (*acr*), Mg^2+ ^transport (*corA*), and the putative tellurium efflux locus *alx*.

The NhaA sodium-proton antiporter is known to contribute to pH regulation and sodium resistance at high pH with aeration [71], whereas the NhaB antiporter is thought to be expressed constitutively. Under oxygen limitation, *nhaA *and *nhaB *showed complex responses to pH and anaerobiosis (Fig. 5). Expression of *nhaA *was higher at both pH extremes than at pH 7, wherease the opposite pattern was seen for *nhaB*, which showed its highest expression at low pH. Under aeration, *nhaB *was up-regulated at pH 5.7.

At least seven multidrug resistance loci showed pH-dependent expression. Multidrug resistance loci up-regulated in acid included *mdtEF*, *mdtG*, *mdtIJ*, and *mdtL *[72,73]. The *mdtEF *locus is part of the *gad-evgA *regulon [74]. For *mdtL*, pH dependence has not been reported in aerated cultures. Base-enhanced or acid-repressed expression was seen for *ampC *[75], *acr *and *emrA*, of which the first two show no response with aeration. Thus, anaerobiosis appeared to increase the overall profile of pH-dependent drug resistance. Besides *ampC*, genes for several other penicillin-binding proteins (PBPs) associated with cell envelope formation (*dac*,*mepA*,*mreBC*) were down-regulated in acid.

### Small regulatory RNAs

The inclusion of intergenic regions (IG) in the Affymetrix probe set revealed regions that express putative small-RNA regulators (sRNA) [76-78]. As shown in Additional file 2, under oxygen limitation, acid up-regulated four IGs that express sRNA molecules known for important roles in environmental response [76,77]. The acid-up-regulated sRNAs included *gadY*, which activates acid-resistance genes *gadWX *[63]; the carbon storage global regulator *csrB *[79]; and two sRNA molecules of unknown function, *ryeA *and *rybC *[76].

### Cross-regulation by other stress conditions

The pH-dependent genes show cross-regulation by various stress factors such as acetate, oxidative stress, and universal stress (annotated in Additional file 1). Anaerobic conditions increased to 197 the number of acetate stress genes up-regulated by acid in log phase (compare Refs. [33-35]). This confirms our prediction that even in early log phase, the small amounts of acetate produced are retained within the cell at high concentration due to the trans-membrane pH difference [34]. Also up-regulated by acid under anaerobiosis were 108 oxidative stress genes up-regulated by H_2_O_2_, paraquat (PQ), or sodium salicylate (Sal) [80,81]. 28 genes were down-regulated (indicated by minus sign, Sal-, PQ-). In addition, 21 acid-dependent genes were identified as universal stress genes [82].

## Discussion and conclusion

Our study showed nearly twice as many pH-dependent genes under anaerobiosis as in aerated cultures [2]. Thus, anaerobiosis appeared to magnify the effects of pH stress response in buffered LBK medium.

Over a hundred "core pH genes" showed parallel response to pH in anaerobic and in aerated cultures. These included genes for envelope maintenance functions, periplasmic proteins, and proton transporters, as well as many genes of unknown function. Further study will determine whether these genes have functions more fundamental to pH homeostasis than do those dependent on oxygen level. In addition, several systems of gene expression responded oppositely to pH with or without aeration, most notably components of electron transport and intermediary metabolism. Thus, central pathways of metabolism showed a surprisingly complex dependence on pH and oxygen.

### Acid stress strongly affects the envelope and membranes

The outer and inner membranes receive direct exposure to external pH. A number of genes encoding outer membrane proteins as well as inner membrane proteins show pH-dependent expression in aerated cultures [2], and many more show pH dependence under oxygen limitation. A gene encoding an inner membrane protein, *yagU*, was identified as a requirement for acid resistance.

The fraction of genes showing pH dependence (with or without aeration) was particularly high in the periplasm, which is fully exposed to external pH due to proton leakage through the outer membrane. As many as half of all periplasmic proteins may show pH-regulated expression. These encode transporters such as AraR, ArtI, and PotD; periplasmic chaperones such as HdeA, HlpA, and FimC; heat-shock protein such as DegP, DegQ; and redox modulators such as DsbA.

Acid appeared to down-regulate the carbon-storage regulon (*csr*) whose effects include activation of flagellar synthesis and biofilm formation. Acid repressed genes encoding two activators of *csrA*, UvrY and LuxR-homolog SdiA [79] but up-regulated an IG that includes an antagonist of *csrA*, the sRNA *csrB*. Down-regulation of csr could be responsible for the decreased flagellar synthesis in acid under anaerobiosis.

### Hydrogenases co-regulated by pH and anaerobiosis

Previous studies of *E. coli *hydrogenases emphasize the differences in environmental response of the different hydrogenase operons [28,29,31,52]. For example Hyd-1 (*hya*) is reported to be up-regulated in acid, but Hyd-2 (*hyb*) is up-regulated in base, under anaerobiosis [28]. Hyd-3 (*hyc*) evolves H_2 _at low pH, whereas Hyd-4 (*hyf*) is active at pH 7.5 [52,83,84]. Another report finds elevation of Hyd-3 and formate dehydrogenase H (Fdh-H) at pH 7.5 [54]. These studies however tested a narrower range of pH than ours, particularly above pH 7. We found a consistent pattern of expression for all five hydrogenase operons (Fig. 5). All hydrogenases showed a high acid/base expression ratio under oxygen limitation, but a low ratio (up-regulated at high pH) in aerobic cultures. The loss of hydrogenase activity in a *hypF *defect eliminated acid resistance of cultures in stationary phase, a finding consistent with the need for hydrogenase expression in an oxygen-limited condition.

It will be of interest to pursue the overall role of hydrogenase activity at low pH versus high pH: Do the hydrogenases generally consume protons, as from formic acid, to reverse acidification; or do they contribute energy gain by splitting hydrogen gas? There is growing evidence for H_2 _as an energy source for *H. pylori *and other pathogens of the digestive tract [85].

### Catabolism and ribosome synthesis are co-regulated by pH and anaerobiosis

A growing number of catabolic enzymes and catabolite transporters are known to be regulated by pH [2,4,86]. Under oxygen limitation, we found additional kinds of catabolism coregulated by pH and oxygen (see Additional file 3). Of particular interest, acid up-regulated the catabolism of sugar derivatives whose fermentation minimized acid production, including sorbitol, glucuronate, and gluconate (Fig. 4).

The large number of catatabolic operons up-regulated by acid in low oxygen was accompanied by dramatic down-regulation of ribosome biosynthesis. The depression of ribosome synthesis may be related to the restricted energy yield of anaerobic metabolism at low external pH, where production of fermentation acids must be limited. Anaerobic growth at low pH may induce a "carbon foraging" strategy similar to that described by Blattner and colleagues [39]. The carbon foraging model states that under conditions where the energy yield of available catabolites is poor, the ribosomal operons are down-regulated and numerous operons for alternative carbon sources are activated. A similar pattern is seen under nitrogen and sulfur starvation [40], where translation and motility are down-regulated, while systems for scavenging nitrogen and sulfur are up-regulated.

The glutamate decarboxylase *gadA *region comprised an anaerobic "pH stress region" of thirteen genes strongly up-regulated by either acid or base compared to pH 7. The *gad *regulon includes the glutamate decarboxylases, *gadA *and *gadBC*, as well as low-pH chaperones *hdeA *and *hdeB *[12,20]. While most studies of *gad *regulation focus on acid, we find that its expression is also up-regulated at high pH, or in LBK medium grown to stationary phase, where pH naturally increases [4,5]. In the present work, at least thirteen ORFs in the *gadA *region showed the same pattern of pH response under anaerobiosis: strong induction in acid compared to pH 7 (ranging from 4-fold to 30-fold increase) with significant response at high pH (2-fold to 8-fold increase). Genes showing pH dependence included the *mdtEF *multidrug resistance locus as well as the outer membrane protein *slp*. Their regulation is known to be mediated by transcription factors GadX-GadW and EvgA-YdeO, as well as by RpoS, H-NS, and cyclic AMP [60,61,74]. The *gad *system enables cells to survive extreme acid [62], but The *gadC *locus is specifically required for cells grown at high pH to survive extreme acid [62]. *Gad *regulon members may also contribute to *E. coli *base resistance, the ability to survive at or above pH 10 [21].

### Multidrug resistance and ion transporters

Under oxygen limitation, acid conditions enhanced expression of many transporters, particularly for metal cations (Table 4). Transport of nickel and iron may be up-regulated in order to acquire nutrients for enzymes needed under acid-anaerobic conditions, such as hydrogenases. On the other hand, silver and copper efflux is up-regulated in order to exclude toxic concentrations of these metals [87]. The solubility and environmental concentrations of these ions is likely to be increased at low pH.

Several proton pumps and cation-proton antiporters up-regulated at high pH showed increased induction at high pH (Figure 5). These included genes encoding the ATPase [2], Na^+^/H^+ ^antiporter *nhaA *[88], and Ca^2+^/H^+ ^antiporter *chaA *(which also functions with sodium). These pumps may enhance uptake or retention of cytoplasmic H^+ ^as pH increases under anaerobiosis, where energetic options are limited. The *nhaB *antiporter [71], however, was down-regulated under anaerobic conditions, and most highly expressed in acid with aeration. NhaB may have a different function from NhaA in pH homeostasis at low pH.

In addition, several multidrug transporters were up-regulated by acid or base, often in association with physiological genes such as *mdtEF *within the *gad *regulon. These drug efflux transporters may have roles in physiology and pH stress resistance that select for their persistence in natural ecosystems [89,90].

### Cross-regulation by other stress factors

An interesting question regarding pH stress is, how much of "pH response" relates directly to pH as opposed to other growth factors, such as stationary phase or starvation-based growth limitation? The doubling rates of our cultures at low pH and high pH were similar, but this represents only one aspect of growth state. Many factors contribute to growth conditions such as stationary phase; for example, both high pH [32] and membrane-permeant acids that depress pH [33-36] are implicated in induction of the RpoS regulon. Starvation for various different nutrients can retard growth by different mechanisms [91] leading to common response patterns such as down-regulated translation and up-regulated scavenging pathways [39,40].

Even at low cell density, moderate acid (pH 6–7) greatly amplifies the uptake of membrane-permeant weak acids such as acetate. In our array analysis, oxygen limitation substantially increased the number of acetate stress genes showing pH-dependent expression (see Additional file 1). Acetate and other permeant acids pass through the bacterial membrane and dissociate in the cytoplasm, causing accumulation of anion and depression of internal pH, inhibiting growth [92]. Growth inhibition by short-chain fatty acids is a significant factor in bacterial colonization of the human colon [93].

## Methods

### Growth conditions

*Escherichia coli *K-12 strain W3110 was obtained originally from Ruth VanBogelen in 1996, and is monitored regularly for RpoS-positive phenotypes including extreme-acid and extreme-base resistance. Bacteria were cultured as for Ref. [2], except that bacteria were grown in closed tubes. Bacteria were cultured in potassium-modified Luria broth (LBK) (10 g/l tryptone, 5 g/l of yeast extract, 7.45 g/l of KCl) buffered with 100 mM homopiperazine-N,N'-bis-2-(ethane-sulfonic acid) (HOMOPIPES) (pK_a _4.55 and 8.12). The pH of the medium was adjusted using KOH to pH 5.7, 7.0, or 8.5. Bacteria were cultured overnight, then diluted 1:1000 into 8.5 ml of buffered medium in an 8.5-ml screw-cap test tube, and incubated at 37°C with slow rotation (8 rpm). Under this condition, oxygen disappears rapidly and anaerobic proteins are highly induced [5,11]. Cultures were grown at 37°C to an optical density (OD_600_) of 0.2. For all cultures, the pH was tested after growth to ensure that the values were maintained at ± 0.2 pH unit of the pH of the original uninoculated medium.

### RNA isolation

Bacterial RNA was stabilized by immediately pouring 8 ml of culture into 16 ml of RNeasy Protect reagent (Qiagen). RNA was isolated as described previously [2] using the RNeasy Kit with on-column DNA digestion (Qiagen), with additional DNA removal using Ambion DNase.

### cDNA preparation and array hybridization

Standard methods were used for cDNA synthesis, fragmentation and end-terminus biotin labeling [2]. Labeled cDNA samples were hybridized to Affymetrix GeneChip *E. coli *Antisense Genome Arrays. Hybridized arrays were stained with streptavidin-phycoerythrin using the Affymetrix Fluidic Station. After staining, arrays were scanned with a GC2500 scanner.

### Expression indices and statistical analysis

Model-based expression analysis was performed on the probe-level data from Affymetrix's DAT files using dChip software [2,41,94]. The model relates target RNA levels to the probe signals by a linear function that weights the significance of all oligo probes for each gene. The data from different arrays were normalized and re-scaled for comparison. Each array was normalized to a baseline array from a pH 7 culture, using local regression on an invariant set of probes [95]. Model-based expression indices were calculated for each gene on each array using only the perfect match probes.

Global relationships among arrays were visualized by performing a principal component analysis [96] on the expression data and plotting arrays in two-dimensional space corresponding to the first two principal components. The gene expression profiles of the arrays were visualized in two-dimensional Euclidian space, by using BRB ArrayTools software v. 3.1 (developed by Richard Simon and Amy Peng Lam).

For each of the three pH conditions, the dataset included five biological replicates (independent with respect to *E. coli *growth, RNA isolation, sample preparation and array hybridization) To test for significant differences in expression between the pH classes, one-way ANOVA was performed on the log_2 _transformed model-based expression indices, on a gene-by-gene basis, at a significance level of 0.001 [2,42]. For all genes in our data set, the median within-group variance was 0.031. Assuming a gene with average within-group variability, our sample size (five replicates for each of three conditions, 7,231 genes and intergenic regions per array) provided statistical power of 98 % to detect a 2-fold difference in gene expression among pH groups. For each gene that displayed significant differences in expression among the classes, pair-wise comparisons of pH classes were determined using Tukey's multiple comparisons procedure to control the family-wise error rate for the T test [2].

To explore categories of differential gene expression, the gene expression profiles of the arrays were visualized in two-dimensional Euclidian space, using BRB ArrayTools software. Categories of differential expression profiles across the pH classes were generated by a hierarchical cluster analysis of differentially expressed genes, based on the average linkage method [97].

### Real-time quantitative RT-PCR

Expression of mRNA for individual genes was quantified by real-time PCR using an ABI Prism7500 DNA analyzer (Applied Biosystems). Primer Express Software v2.0 (Applied Biosystems) was used for primer design. The primers chosen had minimal GC content and amplified 50–70 bp segments of the target genes. The SYBR Green PCR One-Step RT-PCR protocol (Applied Biosystems) was used, in which cDNA reverse transcription and PCR amplification occur in the same well. Nucleic acid concentrations were: 0.1 nM forward primer, 0.1 nM reverse primer, and 50 ng target RNA. PCR cycling conditions were: reverse transcription at 48°C for 30 min, 95°C for 10 min, 40 cycles of denaturation at 92°C for 15 s, and extension at 60°C for 1 min. For detection of primer dimerization or other artifacts of amplification, a dissociation curve was run immediately after completion of the real-time PCR. Individual gene expression profiles were normalized based on measurement of the original RNA sample amplified. All expression levels are presented relative to the expression at pH 7.0 in aerated cultures.

### Strain construction and extreme acid resistance

Strain construction was performed by phage P1 transduction [98]. Mutant alleles containing a kanamycin resistance insertion (Km^R^) were transduced into an isolate of strain W3110 exhibiting strong acid resistance (stationary-phase survival at pH 2). *E. coli *strains were tested for acid resistance by exposure of stationary-phase cultures at pH 2.0 [98]. Cultures were grown from a colony inoculated in LBK medium buffered with HOMOPIPES at pH 5 and incubated overnight at 37°C for 16 h. The overnight cultures were diluted 200-fold in LBK adjusted to pH 2, and incubated 2 h at 37°C. Serial dilutions were plated on LBK, and compared to plated dilutions of the original culture in medium at pH 7. Six plates from six independent cultures were obtained for each condition. Error values represent the standard error of the mean (SEM, n = 6).

### Culture acidification

*E.coli *strain W3110 was grown to stationary phase in closed tubes without headspace, containing half-strength LBK medium supplemented with one of the following carbon sources at 20 mM: glucose, sorbitol, potassium gluconate, or potassium glucuronate. Media were buffered with 5 mM HOMOPIPES, adjusted with KOH to pH 5.0 or pH 8.5. Overnight culture was diluted 500-fold into 8.5 ml of buffered medium in tubes without headspace, and rotated slowly at 37°C for 24 hours. The sorbitol-supplemented cultures were rotated for 48 hours due to their slow growth rate. After growth, the pH was measured. Change in pH was converted to net acid equivalents produced or consumed, based on a standard curve of HCl or KOH added to the original buffered medium.

### Hydrogenase assay

Hydrogenase activity was observed by the methylviologen assay, based on the method of [99]. Stationary-phase cultures in LBK medium were washed and resuspended in 5 mM K_2_HPO_4 _pH 7, 5 mM cysteine, 10 mM benzylviologen, and sealed under hydrogen gas. Purple color change was measured to indicate wild-type hydrogenase activity.

## Authors' contributions

ETH designed the microarray analysis, conducted the experiments and analyzed results, and wrote the first draft manuscript, in consultation with JLS. JCW contributed experiments, produced figures, and helped draft the manuscript. PS constructed and tested the hydrogenase mutant. EY and DPT contributed experiments. MDR and BDJ conducted statistical analysis. SSB supervised the array hybridizations. JLS finalized the analysis and completed the manuscript. All authors have read and approved the final manuscript.

## Supplementary Material

Additional File 1 Supplementary Table 1. Expression indices and expression ratios for pH-dependent genes. Click here for file

Additional File 2 Supplementary Table 2. Expression indices and expression ratios for intergenic regions. Click here for file

Additional File 3 Expression ratios for catabolism and respiration showing anaerobic pH dependence. Click here for file

## Figures and Tables

**Table 1:** Acid/base expression ratios under oxygen limitation (4-fold or higher)

**Acid up-regulated: log_2 _ratio**						**Base up-regulated: log_2 _ratio**					
**pH 5.7/7.0**		**pH 7.0/8.5**		**pH 5.7/8.5**		**pH 5.7/7.0**		**pH 7.0/8.5**		**pH 5.7/8.5**	

*gadA*	**4.89**	*tdcB*^2^	**3.17**	*hycA*	**4.75**	*ompF*^2^	**-3.71**	*gadA*	**-3.46**	*cpxP*^2^	**-4.32**
*aidB*	**4.56**	*srlA*^1^	**3.16**	*adiC*	**4.52**	*proV*^2^	**-2.77**	*cpxP*^2^	**-3.29**	*b3913*	**-4.07**
*yhiM*	**4.55**	*tdcD*	**3.15**	*hycE*	**3.70**	*yfdI*^1^	**-2.72**	*b3913*^2^	**-3.14**	*sdhC*^1^	**-3.36**
*adiC*	**4.10**	*srlB*^1^	**3.06**	*adiA*	**3.67**	*borD*^2^	**-2.71**	*bdm*	**-2.98**	*mutL*^2^	**-3.17**
*sufB*	**3.84**	*cadB*	**3.02**	*citE*	**3.60**	*sdaC*^2^	**-2.57**	*rmf*^2^	**-2.57**	*proV*^2^	**-3.11**
*gadC*	**3.73**	*mdtJ*^1^	**2.91**	*yagU*^1^	**3.60**	*sdhD*^1^	**-2.47**	*gadB*	**-2.50**	*borD*^2^	**-2.94**
*adiA*	**3.67**	*srlD*^1^	**2.87**	*citD*	**3.49**	*nmpC*^2^	**-2.40**	*gadC*	**-2.44**	*yfdI*^1^	**-2.66**
*sufC*	**3.61**	*srlE*^1^	**2.86**	*hydN*	**3.41**	*b2974*	**-2.28**	*gadE*	**-2.23**	*yccA*^2^	**-2.64**
*slp*	**3.47**	*lacA*	**2.53**	*yeiA*	**3.38**	*pppA*^2^	**-2.10**	*rcsA*^1^	**-2.23**	*ymgB*	**-2.62**
*cbpM*	**3.45**	*tdcC*	**2.50**	*gatD*^1^	**3.29**			*ymgB*	**-2.19**	*ymgD*	**-2.49**
*gadB*	**3.21**	*gatD*^1^	**2.45**	*gatC*^1^	**3.12**			*ycfS*^2^	**-2.17**	*rcsA*^1^	**-2.43**
*narH*	**3.20**	*fimA*^1^	**2.38**	*yeiT*	**3.12**			*yjbJ*	**-2.16**	*ycfS*^2^	**-2.42**
*cbpA*	**3.19**	*hycA*	**2.30**	*cadB*	**3.08**			*osmB*^2^	**-2.12**	*dctA*	**-2.41**
*gadE*	**3.19**	*cadA*^1^	**2.28**	*adiY*	**3.06**			*glpD*	**-2.05**	*tgt*^2^	**-2.39**
*mdtE*	**3.17**	*fimC*^1^	**2.18**	*cadA*^1^	**3.06**			*katE*	**-2.01**	*yeaC*^1^	**-2.35**
*narG*	**3.12**	*ykgA*^1^	**2.09**	*mdtJ*^1^	**3.02**			*mutL*^2^	**-1.99**	*yebE*^2^	**-2.31**
*yegP*	**3.07**	*argO*^1^	**2.08**	*aidB*	**3.01**					*htpX*^2^	**-2.27**
*ybaS*	**2.88**	*tdcF*	**2.05**	*yhiM*	**2.89**					*malM*	**-2.24**
*cfa*^1^	**2.83**	*nrfB*	**2.05**	*appC*	**2.88**					*mltD*	**-2.22**
*hyaF*	**2.82**	*ompF*^2^	**2.04**	*appB*	**2.75**					*oppB*^1^	**-2.19**
*ygeW*	**2.82**	*yagU*^1^	**2.03**	*ygeW*	**2.73**					*aceA*	**-2.15**
*gadW*	**2.70**	*gatC*^1^	**2.03**	*ykgA*^1^	**2.68**					*bdm*	**-2.14**
*citE*	**2.69**			*nrfB*	**2.62**					*b1172*	**-2.07**
*gadX*	**2.66**			*hyaF*	**2.58**					*fdoG*^1^	**-2.04**
*citD*	**2.65**			*yccD*	**2.55**					*ompT*^1^	**-2.03**
*ybeL*	**2.65**			*gatA*^1^	**2.46**						
*fbaB*	**2.62**			*hycD*	**2.45**						
*dctR*	**2.60**			*gatB*^1^	**2.37**						
*mdtF*	**2.58**			*appY*	**2.33**						
*narJ*	**2.58**			*appA*	**2.32**						
*sufA*	**2.55**			*gatR*	**2.32**						
*yccJ*	**2.54**			*nemA*^1^	**2.31**						
*ybaT*	**2.49**			*yjdK*	**2.31**						
*dps*^1^	**2.47**			*cfa*	**2.27**						
*hyaD*	**2.47**			*hyaD*	**2.27**						
*hycA*	**2.45**			*hyaA*	**2.26**						
*appB*	**2.44**			*cbpA*	**2.23**						
*appC*	**2.44**			*uspD*^1^	**2.23**						
*yjbJ*	**2.42**			*lysC*^1^	**2.20**						
*hyaC*	**2.41**			*hycF*	**2.19**						
*flgD*^1^	**2.40**			*gatZ*	**2.16**						
*wrbA*	**2.39**			*hyaC*	**2.16**						
*adiY*	**2.37**			*hyaB*	**2.14**						
*hyaE*	**2.36**			*hycB*	**2.11**						
*yhcO*	**2.36**			*nrfA*	**2.06**						
*sufD*	**2.35**			*srlD*^1^	**2.06**						
*hyaA*	**2.33**			*hyaE*	**2.02**						
*hyaB*	**2.32**			*yiiS*^1^	**2.02**						
*uspB*	**2.32**										
*yeiA*	**2.32**										
*hdeD*	**2.30**										
*yjbQ*^1^	**2.30**										
*yeiT*	**2.28**										
*ivy*	**2.26**										
*yhiD*	**2.26**										
*yjjU*^1^	**2.26**										
*appA*	**2.24**										
*yebV*	**2.24**										
*ybhE*	**2.20**										
*psiF*^2^	**2.19**										
*sra*^1^	**2.13**										
*hycE*	**2.10**										
*yqjE*	**2.08**										
*yehE*	**2.06**										
*ydiH*^1^	**2.03**										
*yqjD*^2^	**2.03**										
*yjdI*	**2.02**										
*hydN*	**2.01**										
*yeaQ*^1^	**2.01**										

^1^Acid-induced with aeration [2]

^2^Base-induced with aeration [2]

**Table 2:** Core pH-dependent genes (both aerobic and oxygen-limited)^1^

**Acid up-regulated**			**Base up-regulated**		
*acnA*	*gatZ*	*rcsF*	*acfD*	*katG*	*tsx*
*apaG*	*gcd*	*rob*	*acrA*	*lamB*	*ubiB*
*argO*	*gdhA*	*rpoE*	*acrR*	*maa*	*uxuA*
*aspC*	*gloA*	*rseA*	*alx*	*malE*	*yahA*
*azoR*	*gnd*	*skp*	*artQ*	*malF*	*yahA*
*b1052*	*groS*	*sodB*	*atpA*	*malG*	*yajC*
*cadA*	*grxB*	*srlA*	*atpC*	*malK*	*ybjK*
*cdd*	*hdeA*	*srlB*	*atpF*	*malM*	*yccA*
*cfa*	*hdeB*	*srlD*	*atpH*	*malP*	*yceI*
*clpB*	*hsdR*	*srlE*	*b1172*	*malQ*	*ycfS*
*crcB*	*hybG*	*srlR*	*b3913*	*malT*	*yciC*
*cyaA*	*idi*	*tas*	*bdm*	*mdoD*	*ydcV*
*dadA*	*kdsA*	*tehB*	*borD*	*mltD*	*ydeH*
*dapB*	*kefB*	*tpiA*	*cpxA*	*mppA*	*yebE*
*deoA*	*lysA*	*uspD*	*cpxP*	*mutL*	*yeeI*
*deoB*	*lysC*	*wrbA*	*cpxR*	*nmpC*	*ygaH*
*deoC*	*lysU*	*yagU*	*cvpA*	*nrdD*	*ygaZ*
*deoD*	*mazG*	*yafE*	*dedA*	*nrdG*	*yghG*
*dhaK*	*mcrB*	*ybaK*	*dnaX*	*ompF*	*yghJ*
*dhaL*	*mdaB*	*ybhB*	*dsbA*	*ompT*	*ygiB*
*dhaM*	*mdtI*	*ybjN*	*fadL*	*osmB*	*ygiC*
*dpiA*	*mdtJ*	*ycfP*	*folP*	*pheA*	*yjdM*
*dps*	*menB*	*ychH*	*ftnB*	*pitA*	*yjfO*
*edd*	*menF*	*ydhM*	*fucR*	*pntA*	*yjhC*
*fbp*	*metK*	*ydiH*	*gcvT*	*potD*	*yjhR*
*fimA*	*metN*	*ydiZ*	*glk*	*ppiA*	*ymgD*
*fimI*	*mrp*	*yeaQ*	*glpF*	*pppA*	*ynfD*
*flgB*	*murI*	*yeiA*	*glpX*	*proV*	
*flgC*	*nadC*	*yfiD*	*gpmM*	*purA*	
*flgD*	*narG*	*ygdQ*	*hemY*	*purH*	
*flgE*	*narH*	*yggE*	*hflK*	*purM*	
*flgF*	*nemA*	*ygiN*	*hflX*	*rdoA*	
*flgG*	*nhaB*	*ygiS*	*hisB*	*recA*	
*fliA*	*npr*	*yhbJ*	*hisC*	*rnk*	
*fliK*	*nupG*	*yheL*	*hisD*	*sdaA*	
*fliL*	*pan E*	*yheN*	*hisF*	*sdaC*	
*fre*	*pdxH*	*yheO*	*hisG*	*sdiA*	
*galP*	*pdxK*	*yhhX*	*hisH*	*slt*	
*gapC*	*pdxY*	*yieE*	*hisI*	*spy*	
*gatA*	*pfkB*	*yieF*	*hisJ_*	*sulA*	
*gatB*	*pgi*	*yieP*	*htpX*	*tatA*	
*gatC*	*pps*	*yiiS*	*ilvG*	*tgt*	
*gatD*	*rbsA*	*yjjU*	*ilvN*	*tnaB*	
*gatR*	*rbsC*	*ykgA*	*insH*	*treB*	
*gatY*	*rbsD*	*yqhD*	*ivbL*	*treC*	

**Acid up-regulated (anaerobic)** **Base up-regulated (aerobic)**			**Base up-regulated (anaerobic)** **Acid up-regulated (aerobic)**		

*cydA*	*nhaA*	*ybjH*	*aceE*	*hslJ*	*rfbC*
*cydB*	*pyrB*	*ydcH*	*aceF*	*icd*	*rfe*
*dsdX*	*rbsB*	*yehU*	*aceK*	*lgt*	*ribA*
*fdhF*	*tdcA*	*ygeW*	*add*	*lpxD*	*rplI*
*glgS*	*tdcB*	*yhaN*	*argZ*	*lysP*	*sdhA*
*hupA*	*tdcC*	*yjeJ*	*aspA*	*mltC*	*sdhB*
*lsrK*	*tdcD*	*yjgB*	*b0725*	*nfsA*	*sdhC*
*manX*	*torR*	*yjiY*	*cyoA*	*ndk*	*sdhD*
*manY*	*udp*	*yodD*	*cyoB*	*nuoA*	*sucA*
*manZ*	*uspF*	*yohK*	*cyoC*	*nuoB*	*sucB*
*narI*	*ybaT*	*yqjD*	*cyoD*	*nuoC*	*sucC*
			*dnaJ*	*nuoE*	*sucD*
			*dnaK*	*nuoF*	*thrC*
			*fdoG*	*nuoG*	*tpx*
			*fdoH*	*nuoH*	*wbbI*
			*fimG*	*nuoI*	*yeaC*
			*galF*	*nuoK*	*yebK*
			*gltA*	*nuoL*	*yeiU*
			*gpmA*	*oppC*	*yfdI*
			*groL*	*oppD*	*yffB*
			*guaB*	*oppF*	*yihG*
			*hha*	*purU*	*ylaC*
			*hsdS*	*rcsA*	*yqeG*

^1^Genes were selected as "core pH-dependent" based on their significant differences in expression between acid and base growth conditions, both under oxygen limitation (this report) and under aeration [2].

**Table 3:** *gad *regulon components showing pH dependence under oxygen limitation.

**Gene**	**Function**	**Log_2 _Expression ratio**		
		**pH 5.7/pH 7.0**	**pH 7.0/pH 8.5**	**pH 5.7/pH 8.5**

**GadA region: 3650–3666 kb**				
*slp*	Outer membrane protein induced after carbon starvation	**3.47**	**-1.82**	**1.64**
*dctR*	Probable repressor of dctA dicarboxylate transporter gene	**2.60**	**-1.00**	**1.59**
*yhiD*	integral membrane protein related to MgtC.	**2.26**	**-1.04**	**1.21**
*hdeB*	Periplasmic chaperone of acid-denatured proteins	**0.79**	**-0.55**	
*hdeA*	Periplasmic chaperone of acid-denatured proteins	**0.74**	**-0.50**	
*hdeD*	Putative membrane transporter, H-NS repressed	**2.30**	**-1.26**	**1.03**
*gadE*	Required for stationary phase-induced, pH 5.5 growth medium-induced, and EvgA-induced acid resistance	**3.19**	**-2.23**	**0.95**
*mdtE*	MdtEF-TolC multidrug resistance efflux RND-type transporter; overexpression resistance to erythromycin, deoxycholate, octane and rhodamine	**3.17**	**-1.59**	**1.58**
*mdtF*	MdtEF-TolC multidrug resistance efflux RND-type transporter; overexpression resistance to erythromycin, deoxycholate, octane and rhodamine	**2.58**	**-0.98**	**1.60**
*gadW*	Positive AraC-type regulator of gadA and gadBC, in absence of gadX; repressor of gadX	**2.70**	**-1.17**	**1.53**
*gadY*	sRNA regulates *gadX *and *gadY*	**3.17**	**-1.38**	**1.80**
*gadX*	Positive transcriptional regulator of gadA and gadBC; repressed by HNS and GadW; stimulated by RpoS	**2.66**	**-1.47**	**1.19**
*gadA*	Glutamate decarboxylase isozyme	**4.89**	**-3.46**	**1.43**
**GadB operon: 1567–1570 kb**				
*gadB*	Glutamate decarboxylase isozyme	**3.21**	**-2.50**	
*gadC*	Glutamate transporter	**3.73**	**-2.44**	**1.29**

**Table 4:** Drug resistance, cytoskeletal and ion transport proteins showing pH dependence under oxygen limitation

**Gene**	**Function**	**Log_2_pH ratio **		
**Acid up-regulated**		**pH 5.7/pH 7.0 **	**pH 7.0/pH 8.5 **	**pH 5.7/pH 8.5 **

*chaB*	Regulator of Na^+^-Ca^+^/H^+ ^antiporter	**0.80**		
*chaC*	Regulator of Na^+^-Ca^+^/H^+ ^antiporter	**0.60**	**-0.38**	
*copA*	Copper-, silver-translocating P-type ATPase	**0.86**	**-0.56**	
*cueR*	Activator of copper-responsive regulon genes cueO and copA	**1.31**	**-0.4**	**0.91**
*cusC*	Silver and copper efflux	**0.44**		**0.63**
*feoA*	ferrous iron transport protein A		**0.82**	**1.22**
*feoB*	ferrous iron transport protein B		**0.41**	**0.53**
*marR*^1^	multiple antibiotic resistance protein		**0.31**	
*mdtE*	MdtEF-TolC multidrug resistance efflux transporter	**3.17**	**-1.59**	**1.58**
*mdtF*	MdtEF-TolC multidrug resistance efflux	**2.58**	**-0.98**	**1.60**
*mdtG*^1^	Multidrug resistance efflux transporter	**0.16**		**0.25**
*mdtI*^1^	multidrug transport protein		**1.16**	**1.16**
*mdtJ*^1^	multidrug transport protein		**2.91**	**3.02**
*mdtL*	Multidrug resistance efflux protein			**0.57**
*mgtA*	Mg2^+ ^transport	**0.48**		**0.63**
*nhaA*	Na^+^/H^+ ^antiporter	**1.93**	**-0.93**	**1.00**
*nhaB*^1^	Na^+^/H^+ ^antiporter		**0.41**	**0.37**
*nikA*	periplasmic binding protein for nickel	**0.7**		**0.84**
*nikB*	nickel transport	**0.50**		**0.56**
*nikC*	nickel transport	**0.70**		**0.86**
*nikD*	ATP-binding protein of nickel transport system	**0.60**		**0.74**
*nikE*	ATP-binding protein of nickel transport system	**0.45**		**0.49**
*prc*	carboxy-terminal protease for penicillin-binding protein 3	**0.32**		**0.37**
				

**Base up-regulated**				

*acrA*	acridine efflux pump	**-0.34**	**-0.35**	**-0.69**
*acrB*	acridine efflux pump			**-0.42**
*acrR*^2^	acrAB operon repressor	**-0.93**		**-1.22**
*alx*^2^	Putative metal transporter, homolog of *terC *(tellurium resistance)		**-0.57**	**-0.52**
*ampC*	beta-lactamase; penicillin resistance; penicillin-binding protein	**-0.24**		**-0.12**
*atpA*^2^	ATP synthase, F1 alpha-subunit			**-0.66**
*atpC*^2^	ATP synthase, F1 epsilon-subunit			**-0.66**
*atpD*^2^	ATP synthase, F1 beta-subunit	**-0.35**	**-0.36**	**-0.71**
*atpF*^2^	ATP synthase, F0 subunit b		**-0.46**	**-0.51**
*atpG*^2^	ATP synthase, F1 gamma-subunit			**-0.66**
*atpH*^2^	ATP synthase, F1 delta-subunit		**-0.42**	**-0.58**
*atpI*^2^	ATP synthase subunit			**-0.53**
*chaA*	Na^+^-Ca^+^/H^+ ^antiporter		**-0.86**	**-1.1**
*corA*	Mg2^+ ^transport, system I	**0.10**		**-0.39**
*cutA*	divalent cation tolerance protein	**-0.27**		**-0.3**
*dacA*	D-alanyl-D-alanine carboxypeptidase, fraction A; penicillin-binding protein 5	**-0.58**		**-0.65**
*dacC*	D-alanyl-D-alanine carboxypeptidase; penicillin-binding protein 6	**0.87**	**-1.16**	
*dacD*	penicillin binding protein 6b	**-1.22**		**-0.90**
*emrA*^1^	multidrug resistance secretion protein	**-0.64**		**-0.11**
*mepA*^1^	murein DD-endopeptidase, penicillin-insensitive	**-0.68**		**-0.19**
*mreB*^2^	regulator of ftsI, penicillin binding protein 3, septation function	**-0.53**		**-0.44**
*mreC*	rod shape-determining protein	**-0.29**		**-0.39**
*napF*	Fe-S ferredoxin-type protein	**-0.59**		**-0.14**
*napG*	ferredoxin-type protein	**-0.39**		**-0.20**
*napH*	ferredoxin-type protein	**-0.37**		**-0.20**

^1^Acid up-regulated with aeration [2]

^2^Base up-regulated with aeration [2]
