# Supplementary material for: Oxygen limitation modulates pH regulation of catabolism and hydrogenases, multidrug transporters, and envelope composition in Escherichia coli K-12
Source: BMC Microbiol. 2006 Oct 6;6:89. doi: 10.1186/1471-2180-6-89 (PMC1626474; doi:10.1186/1471-2180-6-89)
Supplement: Additional File 3 — Expression ratios for catabolism and respiration showing anaerobic pH dependence. [file 1471-2180-6-89-S3.doc]

| **TABLE 3. Catabolism and respiration showing anaerobic pH dependence** | | | | | | |
| --- | --- | --- | --- | --- | --- | --- |
| **Group** | **Gene** | **Function** | **Log_2_ Expression ratio** | | |  |
|  |  |  | **pH 5.7/ pH 7.0** | **pH 7.0/ pH 8.5** | **pH 5.7/**  **pH 8.5** |  |
| **Sugar catabolism and TCA cycle** | **Acid up-regulated** | |  |  |  |  |
|  | *acnA^1^* | aconitate hydrase 1 | **0.51** | **-0.45** |  |  |
|  | *araA* | L-arabinose isomerase | **1.44** |  | **1.72** |  |
|  | *araC* | transcriptional regulator for ara operon | **-0.5** |  | **-0.48** |  |
|  | *dhaK^1^* | dihydroxyacetone kinase |  | **0.54** | **0.49** |  |
|  | *dhaL^1^* | dihydroxyacetone kinase |  | **0.41** | **0.46** |  |
|  | *fbaA* | Fructose-bisphosphate aldolase | **0.78** |  |  |  |
|  | *fbaB* | fructose-bisphosphate aldolase | **2.62** | **-1.9** | **0.72** |  |
|  | *fbp* | fructose-1,6-bisphosphatase | **0.47** |  | **0.31** |  |
|  | *frdA* | fumarate reductase |  | **0.4** | **0.58** |  |
|  | *frdB* | fumarate reductase |  | **0.54** | **0.7** |  |
|  | *frdC* | fumarate reductase |  | **0.46** |  |  |
|  | *frdD* | fumarate reductase |  | **0.47** | **0.57** |  |
|  | *fucA* | L-fuculose-1-phosphate aldolase |  | **0.62** | **0.95** |  |
|  | *fucI^2^* | L-fucose isomerase | **-0.36** | **0.6** |  |  |
|  | *galP* | galactose-proton symport of transport system | **0.64** | **0.63** | **1.27** |  |
|  | *gatA^1^* | PTS family enzyme IIA, galactitol-specific | **0.92** | **1.53** | **2.46** |  |
|  | *gatB^1^* | PTS family enzyme IIB, galactitol-specific | **0.81** | **1.56** | **2.37** |  |
|  | *gatC^1^* | PTS family enzyme IIC, galactitol-specific | **1.09** | **2.03** | **3.12** |  |
|  | *gatD^1^* | galactitol-1-phosphate dehydrogenase | **0.84** | **2.45** | **3.29** |  |
|  | *gatR* | split galactitol utilization operon repressor | **0.65** | **1.67** | **2.32** |  |
|  | *gatY^1^* | tagatose-bisphosphate aldolase 1 | **0.71** | **0.85** | **1.56** |  |
|  | *gatZ^1^* | putative tagatose 6-phosphate kinase 1 | **0.92** | **1.24** | **2.16** |  |
|  | *gcd* | glucose dehydrogenase | **0.74** |  | **0.75** |  |
|  | *glgC* | glucose-1-phosphate adenylyltransferase | **0.97** | **-0.64** |  |  |
|  | *gnd^1^* | gluconate-6-phosphate dehydrogenase | **0.56** |  | **0.42** |  |
|  | *gntR* | regulator of gluconate (gnt) operon | **0.3** |  | **0.42** |  |
|  | *gntT^2^* | high-affinity transport of gluconate / gluconate permease | **0.67** |  |  |  |
|  | *gutM* | glucitol operon activator | **-1.49** | **1.6** |  |  |
|  | *malX* | PTS system, maltose and glucose-specific II ABC |  | **0.94** | **0.68** |  |
|  | *manX* | PTS enzyme IIAB, mannose-specific |  | **0.94** | **1.17** |  |
|  | *manY* | PTS enzyme IIC, mannose-specific |  | **1.1** | **1.34** |  |
|  | *manZ* | PTS enzyme, maltodextrin phosphorylase |  | **1.59** | **1.75** |  |
|  | *melA* | alpha-galactosidase; part of glucose catabolism |  | **0.65** | **0.37** |  |
|  | *melR* | regulator of melibiose operon |  | **0.43** | **0.54** |  |
|  | *mtlA* | mannitol PTS permease |  | **0.23** | **0.39** |  |
|  | *mtlD* | mannitol-1-phosphate dehydrogenase | **0.97** |  | **0.98** |  |
|  | *mtlR* | repressor for mtl | **0.82** |  | **0.81** |  |
|  | *pfkB^1^* | 6-phosphofructokinase II; suppressor of pfkA | **1.61** | **-0.98** | **0.63** |  |
|  | *pflB^1^* | formate acetyltransferase 1 |  |  | **0.60** |  |
|  | *pgi^1^* | glucosephosphate isomerase | **0.90** | **-0.41** | **0.49** |  |
|  | *ptsG^1^* | glucose-specific PTS permease |  | **0.7** |  |  |
|  | *ptsO^1^* | phosphocarrier protein HPr-like NPr, nitrogen related, exchanges phosphate with Enzyme I, Hpr | **0.40** |  |  |  |
|  | *rbsA* | ATP-binding component of D-ribose high-affinity transport system |  | **0.36** | **0.45** |  |
|  | *rbsB* | D-ribose periplasmic binding protein | **0.94** |  | **0.87** |  |
|  | *rbsC* | D-ribose high-affinity transport system | **0.78** | **-0.66** |  |  |
|  | *rbsD* | D-ribose high-affinity transport system |  | **0.97** | **1.31** |  |
|  | *sfsA* | probable regulator for maltose metabolism | **0.84** |  |  |  |
|  | *srlA^2^* | PTS system, glucitol/sorbitol-specific IIC component | **-1.85** | **3.16** | **1.31** |  |
|  | *srlB^2^* | PTS family enzyme IIA, glucitol/sorbitol-specific | **-1.46** | **3.06** | **1.61** |  |
|  | *srlD^2^* | glucitol (sorbitol)-6-phosphate dehydrogenase | **-0.81** | **2.87** | **2.06** |  |
|  | *srlE^1^* | PTS system, glucitol/sorbitol-specific | **-1.76** | **2.86** | **1.1** |  |
|  | *srlR^1^* | regulator for gut (srl), glucitol operon | **-0.53** | **0.74** |  |  |
|  | *tpiA^1^* | triosephosphate isomerase | **1.74** | **-0.71** | **1.03** |  |
|  | *ybaY* | glycoprotein/polysaccharide metabolism |  | **-0.58** |  |  |
|  | **Base up-regulated** | |  |  |  |  |
|  | *aceE^1^* | pyruvate dehydrogenase | **0.41** | **-1.48** | **-1.07** |  |
|  | *aceF^2^* | pyruvate dehydrogenase | **0.06** | **-0.88** | **-0.82** |  |
|  | *aceK^1^* | isocitrate dehydrogenase kinase/phosphatase | **-0.28** | **-0.40** | **-0.68** |  |
|  | *araF* | L-arabinose-binding periplasmic protein | **-0.29** |  | **-0.24** |  |
|  | *araH* | high-affinity L-arabinose transport system | **-1.41** |  | **-1.08** |  |
|  | *dld^1^* | D-lactate dehydrogenase |  | **-0.67** |  |  |
|  | *fdoG* | formate dehydrogenase-O | **-1.65** |  | **-2.04** |  |
|  | *fdoH* | formate dehydrogenase-O | **-1.51** |  | **-1.89** |  |
|  | *fdoI* | formate dehydrogenase | **-0.91** |  | **-1.05** |  |
|  | *fruA* | PTS family enzyme IIB'BC, fructose-specific | **-0.57** |  | **-0.6** |  |
|  | *fruB* | PTS family enzyme IIA ; FPr , fructose-specific | **-0.82** |  |  |  |
|  | *fruK* | fructose-1-phosphate kinase | **-0.42** |  | **-0.43** |  |
|  | *fucR^2^* | positive regulator of the fuc operon | **-0.67** |  | **-0.96** |  |
|  | *galF^2^* | homolog of Salmonella UTP--glucose-1-P uridyltransferase | **-0.73** |  | **-0.89** |  |
|  | *glpB^2^* | sn-Glycerol-3-phosphate dehydrogenase | **0.54** | **-0.69** |  |  |
|  | *glpC^2^* | sn-glycerol-3-phosphate dehydrogenase |  | **-0.78** |  |  |
|  | *glpX^2^* | fructose 1,6-bisphosphatase II |  | **-0.34** | **-0.58** |  |
|  | *gltA^1^* | citrate synthase | **-0.59** | **-0.94** | **-1.53** |  |
|  | *gntK* | gluconate kinase 2 in GNT I system | **-0.37** |  |  |  |
|  | *gpmA^1^* | phosphoglyceromutase 1 |  | **-1.27** | **-1.10** |  |
|  | *icdA^1^* | Isocitrate dehydrogenase |  | **-0.59** | **-0.57** |  |
|  | *malE^2^* | maltose transport protein, chemotaxis |  | **-0.66** | **-1.04** |  |
|  | *malF^2^* | maltose transport protein | **-0.81** |  | **-1.02** |  |
|  | *malG^2^* | maltose transport protein | **-0.84** |  | **-1.01** |  |
|  | *malK^2^* | ATP-binding component of transport system for maltose | **-1.68** |  | **-1.96** |  |
|  | *malM^2^* | periplasmic protein of mal regulon | **-1.87** |  | **-2.24** |  |
|  | *malP^2^* | maltodextrin phosphorylase; involved in glycogen degradation |  | **-0.86** | **-1.08** |  |
|  | *malQ^2^* | 4-alpha-glucanotransferase |  | **-0.6** | **-0.78** |  |
|  | *malT^2^* | positive regulator of mal regulon | **-0.65** |  | **-0.81** |  |
|  | *mglB* | galactose-binding transport protein; receptor for galactose taxis | **-0.53** |  | **-0.58** |  |
|  | *sucA^1^* | 2-oxoglutarate decarboxylase |  |  | **-1.03** |  |
|  | *sucB^1^* | 2-oxoglutarate dehydrogenase |  | **-0.69** | **-1.21** |  |
|  | *sucC^1^* | succinyl-CoA synthetase, beta subunit | **-0.55** | **-0.45** | **-0.99** |  |
|  | *sucD^1^* | succinyl-CoA synthetase, alpha subunit |  | **-0.46** | **-0.80** |  |
|  | *treB^2^* | PTS system enzyme II, trehalose specific | **-0.71** |  | **-0.57** |  |
|  | *treC^2^* | trehalase 6-P hydrolase | **-1.22** | **0.41** | **-0.81** |  |
|  | *uxuA* | mannonate hydrolase | **-0.99** | **0.82** |  |  |
| **Proton/ electron transport chain** | **Acid up-regulated** | |  |  |  |  |
|  | *cydB^2^* | cytochrome d terminal oxidase polypeptide |  | **0.69** |  |  |
|  | *frdA^2^* | fumarate reductase |  | **0.40** | **0.58** |  |
|  | *frdB* | fumarate reductase |  | **0.54** | **0.70** |  |
|  | *frdC^2^* | fumarate reductase |  | **0.46** |  |  |
|  | *frdD* | fumarate reductase |  | **0.47** | **0.57** |  |
|  | *fumB* | fumarate hydratase Class I | **-0.77** | **1.76** | **0.99** |  |
|  | *hyaA* | hydrogenase-1 small subunit | **2.33** |  | **2.26** |  |
|  | *hyaB* | hydrogenase-1 large subunit | **2.32** |  | **2.14** |  |
|  | *hyaC* | probable Ni/Fe-hydrogenase 1 b-type cytochrome subunit | **2.41** |  | **2.16** |  |
|  | *hyaD* | processing of HyaA and HyaB proteins | **2.47** |  | **2.27** |  |
|  | *hyaE* | processing of HyaA and HyaB proteins | **2.36** |  | **2.02** |  |
|  | *hyaF* | nickel incorporation into hydrogenase-1 proteins | **2.82** |  | **2.58** |  |
|  | *hybA* | hydrogenase-2 small subunit |  | **1.26** | **0.82** |  |
|  | *hybB* | probable cytochrome Ni/Fe component of hydrogenase-2 | **-0.94** | **1.15** |  |  |
|  | *hybC* | probable large subunit, hydrogenase-2 | **-1.12** | **1.22** |  |  |
|  | *hybD* | probable processing element for hydrogenase-2 | **-0.9** | **1.07** |  |  |
|  | *hybE* | member of hyb operon | **-0.86** | **1.09** |  |  |
|  | *hybF* | may modulate levels of hydrogenease-2 | **-0.72** | **0.99** |  |  |
|  | *hybG* | hydrogenase-2 operon protein | **-0.55** | **1.15** | **0.6** |  |
|  | *hybO* | hydrogenase-2, small chain |  | **1.19** | **0.84** |  |
|  | *hycA* | transcriptional repression of hyc and hyp operons | **2.45** | **2.3** | **4.75** |  |
|  | *hycB* | hydrogenase-3, Fe-S subunit | **1.4** | **0.71** | **2.11** |  |
|  | *hycC* | membrane-spanning protein of hydrogenase 3 | **0.45** |  | **0.74** |  |
|  | *hycD* | membrane-spanning protein of hydrogenase 3 | **1.64** | **0.82** | **2.45** |  |
|  | *hycE* | hydrogenase 3, large subunit | **2.1** | **1.6** | **3.7** |  |
|  | *hycF* | probable iron-sulfur protein of hydrogenase 3 | **1.59** | **0.6** | **2.19** |  |
|  | *hycG* | hydrogenase activity | **1.42** | **0.51** | **1.93** |  |
|  | *hycH* | processing of large subunit of hydrogenase 3 | **0.69** | **0.36** | **1.06** |  |
|  | *hycI* | protease involved in processing C-terminal end of hydrogenase 3 | **0.81** |  | **1.14** |  |
|  | *hydH* | sensor kinase for HydG, hydrogenase 3 activity |  | **0.32** |  |  |
|  | *hydN* | involved in electron transport from formate to hydrogen | **2.01** | **1.39** | **3.41** |  |
|  | *hypA* | pleiotrophic effects on 3 hydrogenase isozymes | **0.74** | **0.71** | **1.46** |  |
|  | *hypB* | guanine-nucleotide binding protein | **0.48** | **0.5** | **0.98** |  |
|  | *hypC* | hydrogenase expression/formation protein |  | **0.59** | **0.89** |  |
|  | *hypD* | hydrogenase expression/formation protein |  | **0.53** | **0.8** |  |
|  | *hypE* | hydrogenase 3 maturation protein | **0.32** | **0.31** | **0.63** |  |
|  | *hypF* | hydrogenase maturation protein |  |  | **0.33** |  |
|  | *mdaA^1^* | Nitroreductase A |  | **-0.29** | **-0.49** |  |
|  | *mdaB* | modulator of drug activity B | **1.13** | **-0.50** | **-0.63** |  |
|  | *narG* | nitrate reductase 1, alpha subunit | **3.12** | **-1.19** | **1.92** |  |
|  | *narH* | nitrate reductase 1, beta subunit | **3.20** | **-1.25** | **1.94** |  |
|  | *narI* | nitrate reductase 1, cytochrome b(NR), gamma subunit | **0.84** |  | **0.79** |  |
|  | *narJ* | nitrate reductase 1, delta subunit | **2.58** | **-0.90** | **1.68** |  |
|  | *narK* | nitrite extrusion protein | **1.25** |  | **1.30** |  |
|  | *narX* | nitrate/nitrate sensor | **0.23** |  | **0.22** |  |
|  | **Base up-regulated** | |  |  |  |  |
|  | *atpA^2^* | membrane-bound ATP synthase |  |  | **-0.66** |  |
|  | *atpC^2^* | membrane-bound ATP synthase |  |  | **-0.66** |  |
|  | *atpD^2^* | membrane-bound ATP synthase | **-0.35** | **-0.36** | **-0.71** |  |
|  | *atpF^2^* | membrane-bound ATP synthase |  | **-0.46** | **-0.51** |  |
|  | *atpG^2^* | membrane-bound ATP synthase |  |  | **-0.66** |  |
|  | *atpH^2^* | membrane-bound ATP synthase |  | **-0.42** | **-0.58** |  |
|  | *atpI^2^* | membrane-bound ATP synthase subunit |  |  | **-0.53** |  |
|  | *cyoA^1^* | Cytochrome o oxidase |  |  | **-0.82** |  |
|  | *cyoD^1^* | cytochrome o ubiquinol oxidase |  | **-0.76** | **-1.30** |  |
|  | *fdnH* | formate dehydrogenase-N | **-1.46** | **0.80** | **-0.66** |  |
|  | *fdnI* | formate dehydrogenase-N | **-1.55** | **0.93** | **-0.62** |  |
|  | *fdoG^1^* | formate dehydrogenase-O | **-1.65** |  | **-2.04** |  |
|  | *fdoH^1^* | formate dehydrogenase-O | **-1.51** |  | **-1.89** |  |
|  | *fdoI* | formate dehydrogenase | **-0.91** |  | **-1.05** |  |
|  | *hyfE* | hydrogenase 4 membrane subunit | **-1.06** |  | **-0.86** |  |
|  | *napF* | Fe-S ferredoxin-type protein, electron transfer | **-0.59** | **0.45** |  |  |
|  | *napG* | ferredoxin-type protein: electron transfer | **-0.39** |  |  |  |
|  | *napH* | ferredoxin-type protein, electron transfer | **-0.37** |  |  |  |
|  | *narP* | nitrate/nitrite response regulator | **-0.92** | **0.54** |  |  |
|  | *narQ* | sensor for nitrate reductase system, protein histidine kinase | **-0.23** |  | **-0.27** |  |
|  | *ndh^1^* | respiratory NADH dehydrogenase | **-0.61** |  |  |  |
|  | *nuoA* | NADH dehydrogenase I chain A | **-0.79** |  | **-0.86** |  |
|  | *nuoB* | NADH dehydrogenase I chain B | **-0.69** |  | **-0.92** |  |
|  | *nuoC^1^* | NADH dehydrogenase I chain C, D | **-0.83** |  | **-1.05** |  |
|  | *nuoE* | NADH dehydrogenase I chain E | **-0.71** |  | **-0.91** |  |
|  | *nuoF* | NADH dehydrogenase I chain F | **-1.00** |  | **-1.40** |  |
|  | *nuoG^1^* | NADH dehydrogenase I chain G | **-0.98** | **-0.56** | **-1.54** |  |
|  | *nuoH^1^* | NADH dehydrogenase I chain H | **-0.91** | **-0.49** | **-1.40** |  |
|  | *nuoI^1^* | NADH dehydrogenase I chain I | **-0.64** | **-0.53** | **-1.17** |  |
|  | *nuoJ^1^* | NADH dehydrogenase I chain J | **-0.67** | **-0.45** | **-1.11** |  |
|  | *nuoK^1^* | NADH dehydrogenase I chain K | **-0.95** | **-0.52** | **-1.46** |  |
|  | *nuoM* | NADH dehydrogenase I chain M | **-0.84** |  | **-1.36** |  |
|  | *nuoN^1^* | NADH dehydrogenase I chain N | **-0.46** |  | **-0.89** |  |
|  | *sdhA^1^* | succinate dehydrogenase |  | **-0.68** | **-1.20** |  |
|  | *sdhB^1^* | succinate dehydrogenase, iron sulfur protein | **-0.70** | **-0.94** | **-1.64** |  |
|  | *sdhC^1^* | succinate dehydrogenase, cytochrome b556 | **-1.86** | **-1.50** | **-3.36** |  |
|  | *sdhD^1^* | succinate dehydrogenase, hydrophobic subunit | **-2.47** |  | **-1.78** |  |
| **Amino acid catabolism/transport** | **Acid up-regulated** | |  |  |  |  |
|  | *adiA* | biodegradative arginine decarboxylase | **3.67** |  | **3.67** |  |
|  | *adiC* | Arginine:agmatine antiporter, arginine-dependent acid resistance | **4.10** |  | **4.52** |  |
|  | *adiY* | putative ARAC-type regulatory protein | **2.37** | **0.69** | **3.06** |  |
|  | *artI^2^* | arginine 3rd transport system | **0.63** | **-0.68** |  |  |
|  | *cadA^1^* | lysine decarboxylase 1 |  | **2.28** | **3.06** |  |
|  | *cadB* | transport of lysine/cadaverine |  | **3.02** | **3.08** |  |
|  | *cadC* | transcriptional activator of cad operon |  | **0.54** |  |  |
|  | *cysT* | Cysteine tRNA |  | **0.96** |  |  |
|  | *dadA^1^* | D-amino acid dehydrogenase subunit | **0.79** | **-0.73** |  |  |
|  | *dppF* | putative ATP-binding component of dipeptide transport system | **0.62** |  |  |  |
|  | *gadA* | Glutamate decarboxylase isozyme | **4.89** | **-3.46** | **1.43** |  |
|  | *gadB* | Glutamate decarboxylase isozyme | **3.21** | **-2.50** |  |  |
|  | *gadC* | Glutamate transporter | **3.73** | **-2.44** | **1.29** |  |
|  | *lysA* | diaminopimelate decarboxylase | **1.13** |  | **1.18** |  |
|  | *lysC^1^* | aspartokinase III, lysine sensitive | **0.51** | **1.69** | **2.20** |  |
|  | *lysP^1^* | lysine-specific permease | **-0.84** | **0.98** |  |  |
|  | *tdcA* | transcriptional activator of tdc operon | **-0.91** | **1.11** |  |  |
|  | *tdcB^2^* | threonine dehydratase, catabolic | **-1.70** | **3.17** | **1.47** |  |
|  | *tdcC* | anaerobically inducible L-threonine | **-1.70** | **2.50** | **0.80** |  |
|  | *tdcD* | putative kinase | **-1.84** | **3.15** | **1.32** |  |
|  | *tdcE* | probable formate acetyltransferase 3 | **-0.82** | **1.45** | **0.62** |  |
|  | *tdcF* |  | **-0.89** | **2.05** | **1.16** |  |
|  | *tdcG* | L-serine deaminase, L-serine dehydratase | **-1.02** | **1.91** | **0.89** |  |
|  | **Base up-regulated** | |  |  |  |  |
|  | *artJ* | arginine 3rd transport system |  |  | **-0.58** |  |
|  | *artM^2^* | arginine 3rd transport system permease protein |  | **-0.37** | **-0.39** |  |
|  | *artP* | ATP-binding component of 3rd arginine transport system |  | **-0.58** |  |  |
|  | *artQ* | arginine 3rd transport system permease protein |  | **-0.54** |  |  |
|  | *potA* | ATP-binding component of spermidine/putrescine transport | **-0.65** |  | **-0.71** |  |
|  | *potB* | spermidine/putrescine transport protein | **-0.78** |  | **-0.72** |  |
|  | *potC* | spermidine/putrescine transport protein | **-0.42** | **-0.40** | **-0.82** |  |
|  | *potD^2^* | spermidine/putrescine periplasmic transport protein | **-0.89** | **-0.51** | **-1.40** |  |
|  | *sdaA^2^* | L-serine deaminase |  | **-0.51** |  |  |
|  | *sdaB^2^* | L-serine dehydratase | **-0.44** | **0.34** |  |  |
|  | *sdaC^2^* | probable serine transporter | **-2.57** | **1.00** | **-1.57** |  |
|  | *speD* | S-adenosylmethionine decarboxylase |  |  | **-1.58** |  |
|  | *speE* | spermidine synthase (putrescine aminopropyltransferase) |  |  | **-1.66** |  |
|  | *tnaA^2^* | tryptophanase | **-0.20** | **0.19** |  |  |
|  | *tnaB^2^* | low affinity tryptophan permease | **-1.22** | **0.78** | **-0.44** |  |

^1^Acid up-regulated with aeration [2]

^2^Base up-regulated with aeration [2]
